# Efficacy and Safety of ‘Fixed Dose’ versus ‘Loose’ Drug Regimens for Treatment of Pulmonary Tuberculosis in Two High TB-Burden African Countries: A Randomized Controlled Trial

**DOI:** 10.1371/journal.pone.0157434

**Published:** 2016-06-20

**Authors:** Abraham Aseffa, Joseph N. Chukwu, Mahnaz Vahedi, Emmanuel N. Aguwa, Ahmed Bedru, Tesfamariam Mebrahtu, Oliver C. Ezechi, Getnet Yimer, Lawrence K. Yamuah, Girmay Medhin, Cathy Connolly, Wasima Rida, Getachew Aderaye, Alimuddin I. Zumla, Philip C. Onyebujoh, 4FDC Study Group

**Affiliations:** 1 Armauer Hansen Research Institute (AHRI), ALERT Compound, Addis Ababa, Ethiopia; 2 German Leprosy and Tuberculosis Relief Association, Hill View, Enugu, Nigeria; 3 Special Programme for Research and Training in Tropical Diseases (WHO/TDR), Avenue Appia, Geneva, Switzerland; 4 University of Nigeria Enugu Campus, Enugu, Nigeria; 5 Division of Clinical Sciences, Nigerian Institute of Medical Research, Yaba, Lagos, Nigeria; 6 Department of Pharmacology, School of Medicine, College of Health Sciences, Addis Ababa University, Addis Ababa, Ethiopia; 7 Aklilu Lemma Institute of Pathobiology, Addis Ababa University, Addis Ababa, Ethiopia; 8 Biostatistics Unit, Medical Research Council, Durban, South Africa; 9 Biostatistics Consultant, Arlington, Virginia, United States of America; 10 Department of Internal Medicine, Addis Ababa University, Addis Ababa, Ethiopia; 11 Division of Infection and Immunity, Centre for Clinical Microbiology, University College London, and NIHR Biomedical Research Centre, UCL Hospitals NHS Foundation Trust, London, United Kingdom; 12 World Health Organization, Inter-country Support Team for East and Southern Africa, Harare, Zimbabwe; The George Washington University School of Medicine and Health Sciences, UNITED STATES

## Abstract

**Background:**

There are limited data on the performance of the use of fixed-dose combination (FDC) TB drugs when used under programmatic settings in high TB-endemic countries. We evaluated the efficacy and safety of FDC versus loose formulation (LF) TB treatment regimens for treatment of pulmonary TB (PTB) in the context of actual medical practice in prevailing conditions within programmatic settings in five sites in two high TB-burden African countries.

**Methods:**

A two-arm, single-blind, randomized clinical trial comparing FDCs with separate LFs involving 1000 adults newly diagnosed with culture positive PTB was conducted at five sites in two African countries between 2007 and 2011. Participants were randomized to receive daily treatment with anti-TB drugs given as either FDC or separate LFs for 24 weeks (intensive phase– 8 weeks of isoniazid, rifampicin, ethambutol and pyrazinamide; continuation phase– 16 weeks of rifampicin and isoniazid). Primary outcome measures were microbiological cure and safety at the end of six months’ treatment; pre-specified non-inferiority margin for difference in cure rate was 4%. The primary efficacy analysis was based on the modified intent to treat (mITT) cohort comprising all randomized patients with a positive baseline culture result for TB and who received at least one dose of study treatment. Patients missing end of treatment culture results were considered failures. Further analyses were done in which mITT patients without an end of treatment (EOT) culture were excluded in a complete case analysis (mITT_cc_) and a per protocol cohort analysis defined as mITT_cc_ patients who received at least 95% of their intended doses and had an EOT culture result.

**Results:**

In the mITT analysis, the cure rate in the FDC group was 86.7% (398/459) and in the LF group 85.2% (396/465) (difference 1.5-% (90% confidence interval (CI) (-2.2%– 5.3%)). Per Protocol analysis showed similar results: FDC 98.9% (359/363) versus LF 96.9% (345/356), (difference 2.0% (90% CI: 0.1%– 3.8%)). The two arms showed no significant differences in terms of safety, early culture conversion and patient adherence to treatment.

**Interpretation:**

The comparison of the two drug regimens satisfied the pre-specified non-inferiority criterion. Our results support the WHO recommendations for the use of FDC in the context of actual medical practice within health services in high TB-endemic countries.

**Trial Registration:**

ISRCTN Registry 95204603

## Introduction

Tuberculosis (TB) remains a major global public health problem [1]. Patient non-adherence to TB treatment regimens and inappropriate prescription of TB drugs are important contributing factors to treatment failure and development of drug resistant strains of *Mycobacterium tuberculosis* [2] which now threaten gains being made in global TB control [3]. The World Health Organization (WHO) and the International Union Against Tuberculosis and Lung Disease (IUATLD/The Union) endorsed the use of Fixed Dose Combination (FDC) drug formulations for treatment of pulmonary TB (PTB) in 1994 [4]. These recommendations were based mainly on expert opinion without any evidence base from randomized clinical trials or systematic evaluation of safety and efficacy at programme level. The underlying assumption was that FDC formulations would improve prescribing, make dispensing easier, simplify TB drug treatment, enable patient acceptability, reduce risk of inappropriate dosing and pill burden, and prevent inadvertent monotherapy arising from physician or patient error [5]. Over the past 2 decades national TB control programmes of most African countries have introduced FDCs for treatment of TB [4,6].

In 1997, concerns about the bioavailability of rifampicin (because of its relatively poor bioavailability when combined with isoniazid) led WHO and the IUATLD to issue further guidelines for ensuring the bioavailability of each drug component of the FDC formulations [7]. Subsequently several clinical trials and observational studies have assessed the effectiveness of FDC in reducing treatment failure, improving patient compliance, disease relapse and emergence of drug resistance. A recent systematic review and meta-analysis of 15 trials [8] showed no benefit to treatment outcomes. Despite conflicting results, the FDC formulations continue to be recommended by WHO [9], the American Thoracic Society [10] and International Standards for Tuberculosis Care [11]. Furthermore, clinical trials conducted under optimal research conditions do not reflect the actual performance of test regimens under conditions of routine programmatic clinical practice. There are scant data on the performance of FDCs in terms of safety and efficacy when used under prevailing health services conditions in sub-Saharan African countries. We thus performed a randomized clinical trial to evaluate the efficacy and safety of FDC versus loose formulation (LF) TB treatment regimens for treatment of pulmonary TB (PTB) in the context of actual medical practice in prevailing conditions within programmatic settings in five sites in two high TB/HIV burden African countries.

## Methods

### Study design and participants

This study was a multi-centre, two-arm, single-blind randomized trial which evaluated the non-inferiority of FDC (test arm) to LF (control arm) TB drugs in newly diagnosed patients with PTB. The drug regimens for this study consisted of 8 weeks of daily, directly observed treatment (DOT) for the intensive phase, followed by 16 weeks of daily DOT (continuation phase) as per national TB programme guidelines.

**Ethical considerations:** The study received approval from the AHRI/ALERT and National Ethical Review committees and the Drug Administration and Control Authority (DACA) in Ethiopia; the University of Nigeria Teaching Hospital, Health Research Ethics Committee in Nigeria; and the WHO Research Ethics Review Committee.

### Eligibility criteria

Inclusion criteria were: adults (>18 years) with newly diagnosed, sputum smear acid-fast bacilli positive, PTB; weight of at least 40kg; a verifiable home address; willingness to have an HIV test; CD4 counts >350cells/mm^3^ if HIV positive; and written informed consent. Exclusion criteria were: HIV-positive patients on antiretroviral treatment; previous history of TB treatment; co-morbidities requiring hospitalization; concomitant immunosuppressive treatment; psychiatric illness, alcohol or drug abuse; pregnancy, and extrapulmonary-TB.

### Study sites

The study was conducted in five sites in the two African countries: Ethiopia–(i) St. Peter’s Hospital, (ii) Bole Health Centre, and (iii) Adama Hospital; Nigeria–(iv) Mile 4 Hospital, and (v) Aba South Health Centre. These two countries were selected following a WHO/TDR-sponsored workshop to develop research capacity of national TB control programmes in limited resource settings. The process entailed a competitive approach that identified the most appropriate countries to design and implement the identified research questions. The protocol design was approved by WHO, prior to selection of countries to conduct the clinical study. The trial was coordinated by the Armauer Hansen Research Institute (AHRI) jointly with the Ministry of Health, Ethiopia and German Leprosy and Tuberculosis Relief Association (GLRA), in conjunction with the Ministry of Health, Nigeria and conducted in January 2007 to April 2011.

### Procedures

2,209 TB suspected patients were screened for pulmonary TB by sputum smear Ziehl-Neelsen stain or fluorescence microscopy. Patients were considered positive for pulmonary TB if they had 2 out of 3 smears positive or 2 out of 5 smear positive in the event that only one of the first three smears was positive. Patients with newly diagnosed PTB were counselled appropriately to ascertain their willingness to participate in the trial and were provided information sheet with written informed consent. If illiterate an independent witness was chosen by individual patient to read the written materials. The next step was to apply inclusion and exclusion criteria, including physical examination and laboratory tests (liver function test, HIV counselling and testing, fasting blood sugar test, full blood count, uric acid, renal function test and urinary pregnancy test for women in reproductive age). At all levels patients were excluded if they did not meet the inclusion criteria. Subsequently, 1,209 enrolled patients were randomly assigned into two arms to receive either LF (control arm) or FDC (test arm) for a total of 24 weeks: intensive phase–8 weeks [56 doses] of daily isoniazid, rifampicin, ethambutol and pyrazinamide; continuation phase: 16 weeks [112 doses] of daily rifampicin and isoniazid. The four FDCs and the two FDCs used were identical across the different study sites and both formulations included rifampicin. The first dose of drugs for each patient was based on the pre-treatment weight; subsequent doses were based on the weight at each follow-up visit. The anti-TB drug preparations were all from the same source (Lupin Pharmaceuticals, India—a WHO-approved pre-qualified source and supplier of TB drugs to the Nigerian and Ethiopian national TB programmes through Global Drug Facility). The names, dose preparations, indications and duration of use of the medications were properly documented.

### Bacteriology

At each visit, two sputum samples were collected and examined for the presence of acid-fast bacilli, either by Ziehl-Neelsen stain or by fluorescence microscopy. Patients were considered positive if they met the following national programme criteria: (i) two out of three consecutive sputum smears are read as positive, or (ii) two out of five are read as positive in the event that only one of the first three smears is positive (in which case the patient will be asked for two more specimens: one morning, one spot). All smears were also cultured on LJ slopes in accordance with WHO guidelines. The proportion method was used to test for susceptibility to the TB drugs used in this study.

### Outcome measures

*Primary outcome measure* was cure at end of 24 weeks treatment (6 months short-course chemotherapy). If week 24 results were unavailable, week 20 was used. Cure was defined as one negative sputum culture in patients who did not fail treatment.

*Secondary outcome measures* were: a) early response rates (proportion of patients with negative culture results at 8 weeks after initiation of therapy); b) proportion of patients with recurrence during the 72 weeks follow up period after the end of treatment; c) proportion cured in HIV positive patients; d) proportion of patients with serious adverse events any time during chemotherapy; e) proportion of patients with any adverse events during chemotherapy; and f) proportion of patients completing treatment.

### Sample size

Sample size for this study was calculated to demonstrate FDCs' non-inferiority to the LF in terms of efficacy in the treatment of TB. To this effect, 498 patients per arm was sufficient to rule out a clinically acceptable 4% decrease in the proportion of patients cured on FDC with 90% power using a one-sided, 5% level Miettinen and Nurminen score test assuming a 96% cure rate in the LF arm and no more than 10% of patients without an EOT culture (who would be excluded from analysis). Thus, 500 participants were randomized to each arm.

Given concerns that a complete case analysis could be biased, the primary analysis was changed such that patients without an EOT culture would be counted as failures. If 10% of patients had missing EOT culture, the cure rate in the LF arm would drop to 86% and study power would decrease to 57%. However, if 5% of patients had missing culture, the LF cure rate would be 91% and power would be about 70%.

### Randomization and masking

Participants were allocated to treatment arms according to a computer-generated randomization list prepared and held by WHO/TDR, the study sponsor. Each centre was provided with a batch of sealed opaque envelopes. These were opened, and drugs dispensed to participants, only by a treatment nurse who was not an investigator in the trial. The treatment nurses kept treatment allocation logs which were not available to the investigators until conclusion of the analysis.

### Statistical analysis

The efficacy analysis used data from the modified intent to treat (mITT) cohort which consisted of all randomised patients who had a positive sputum culture result at baseline and who had taken at least one dose of study treatment. Those mITT patients who had an unknown sputum culture result at end of treatment (EOT) were assumed to be failures. This efficacy analysis is denoted by mITT.

The number of participants cured at week 24 was tabulated for each trial arm and expressed as percentages. The difference between study groups was tested using the Agresti-Caffo method [12] to calculate a lower limit one-sided 95% confidence interval (or its equivalent—the lower limit of a two-sided 90% CI). The FDC arm was considered non-inferior to the LF if the lower limit of the CI for the risk difference (% FDC cured–%LF cured) did not extend below -4%.

Two further analyses were performed. The first used the per protocol cohort (PP), a subset of the mITT patients who had completed treatment and had a known sputum culture result at end of treatment. Treatment adherence was defined as those who took at least 54/56 (96.4%) doses in the intensive phase and at least 107/112 (95.5%) doses in the continuation phase. The second analysis was a complete case scenario where mITT patients who had an unknown sputum culture result at end of treatment (EOT) were excluded. These efficacy analyses are referred to as PP and mITTcc.

Seven secondary outcomes were analysed and treatment arms compared: (i) conversion at week 8 (early response) using two cohorts (mITT and PP), (ii) treatment completion using ITT cohort, (iii) cure among HIV+ patients using ITT cohort, (iv) occurrence of any drug related adverse events stratified by treatment phase and overall using ITT cohort, (v) occurrence of any serious adverse event stratified by treatment phase and overall using ITT cohort, (vi) occurrence of death stratified by treatment phase and over all using ITT cohort and (vii) relapse among those cured at week 24. For each of these outcomes, difference in the proportion of the occurrence of the target event between the treatment FDC arm and LF arm were calculated. Pearson’s chi-square test with continuity correction was used to assess the statistical significance. The exception was relapse for which the finding was described in the narrative.

### Data access and guarantee

Joseph N. Chukwu and Abraham Aseffa had full access to all the data in the study. Abraham Aseffa had final responsibility for the decision to submit for publication.

## Results

**Fig 1:**
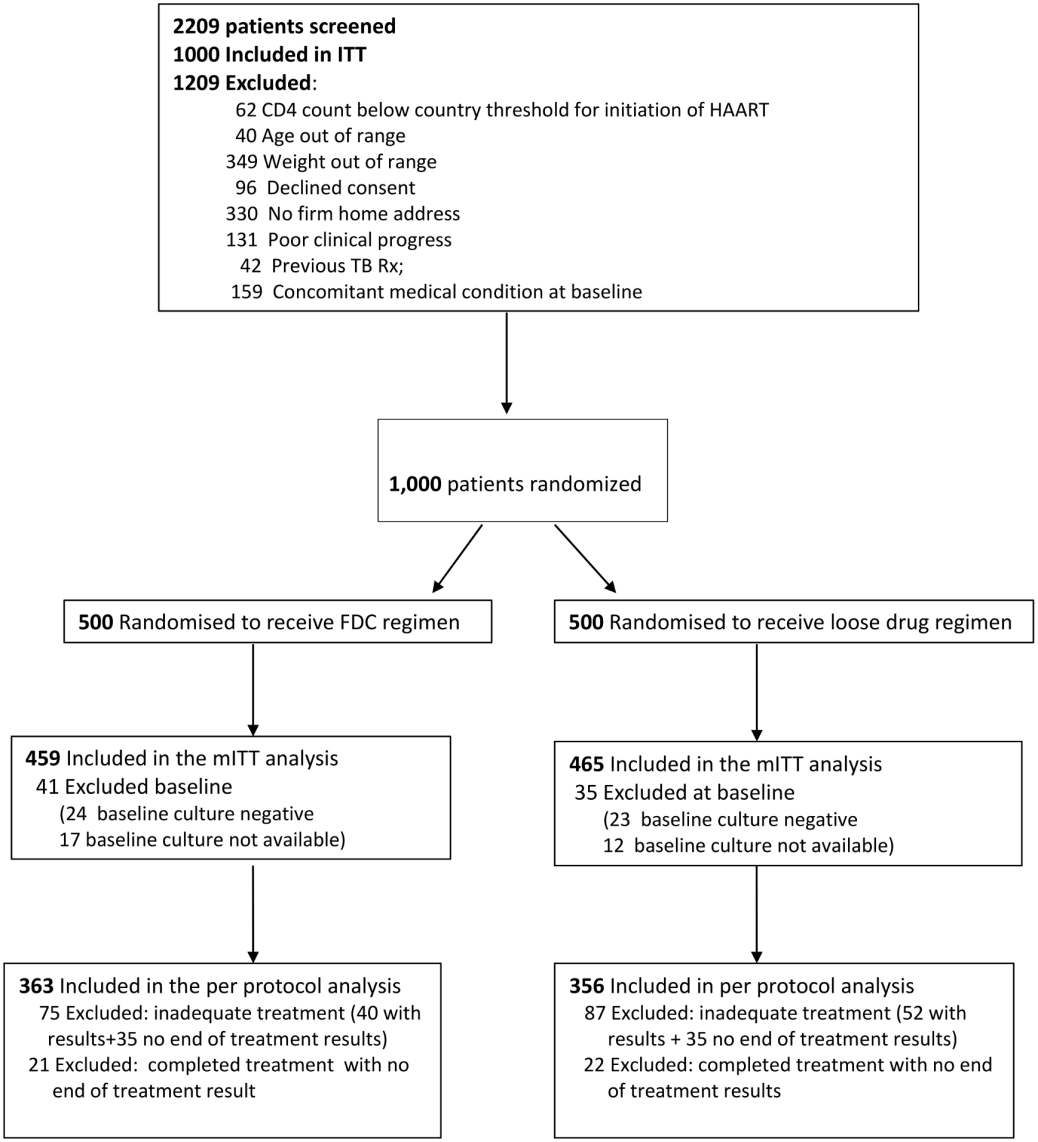
Flow Chart showing recruitment and follow-up during treatment period.

The study enrolled and randomized a total of 1000 patients (500 in FDC arm; 500 in LF arm) between 2007 and 2010, and completed follow-up in 2011 (Fig 1). The mITT analysis included 924 participants (FDC arm: 459/500 [91.8%]; LF arm: 465/500 [93.0%]). Excluded participants included 76 who had positive culture result at baseline (41 in FDC arm and 35 in the LF arm). The per protocol (PP) cohort was a subset of the mITT and excluded a further 162 participants with inadequate treatment (75 FDC and 87 LF), plus 43 patients who completed treatment but without a known EOT culture result (21 FDC and 22 LF). Thus, the PP analysis was based on 719 patients (FDC arm: 363/459 [79.1%]; LF arm: 356/465 [76.6%]).

Baseline characteristics of mITT study participants were similar in the two groups (Table 1). The majority of study participants (79.3%) were less than 40 years of age; 57.4% were males, 17.1% had noticeable symptoms of TB for more than 24 weeks, only 5.4% were HIV positive, and 1.4% had concurrent diseases.

**Table 1:** Baseline Characteristics of mITT Population.

	Treatment arm					
	FDC Arm		LF ARM		Total	
	No.	%	No.	%	No.	%
**Study country**						
Ethiopia	239	52	242	52	481	52
Nigeria	220	48	223	48	443	48
**Age (yr)**						
<25	173	38	172	37	345	37
25-39	185	40	203	44	388	42
> = 40	101	22	90	19	191	21
**Sex**						
Female	188	41	206	44	394	43
Male	271	59	259	56	530	57
**Weight (Kg)**						
40–45	130	28	129	28	259	28
46–55	281	61	280	60	561	61
56–66	39	8	51	11	90	10
67+	9	2	5	1	14	2
**BMI (kg/m2)**						
Underweight <18.5	299	65	292	63	591	64
Overweight (26.0–29.9)	2	0	0	0	2	0
Obese (> = 30.0)	0	1	0	1	0	0
Healthy(18.5–25.9)	158	34	172	37	330	36
**Duration of TB symptoms**						
<3 weeks	10	2	13	3	23	2
-6 wks	64	14	63	14	127	14
7–12 wks	183	40	183	39	366	40
12–24 wks	136	30	114	25	250	27
>24 wks	66	14	92	20	158	17
**Haemoglobin (g/dL)**						
<8.0	2	0	8	2	10	1
> = 8.0	457	100	457	98	914	99
**HIV test**						
Positive	20	4	30	6	50	5
Negative	438	95	435	94	873	94
Not available	1	0	0	0	1	0
**CD4 Count**						
<350	4	20	6	20	10	20
> = 350	16	80	24	80	40	80
**Concurrent disease at baseline**						
Yes	7	2	6	1	13	1
No	452	98	459	99	911	99

### Treatment adherence

Treatment adherence was defined as at least 54/56 (96.4%) doses taken in the 8 week intensive phase and at least 107/112 (95.5%) doses taken in the 16 week continuation phase. Among the total randomised population (ITT), compliance was very high. In the intensive phase 96.6% (966/1000) completed treatment; 97.0% (485/500) in the FDC arm and 96.2% (481/500) in the LF arm (p = 0.6). Similarly, 80.7% (807/1000) of the total study participants completed treatment in the continuation phase (FDC: 81.6% (408/500) vs LF:79.8% (399/500), p = 0.5). Of the mITT cohort, 82.5% (762/924) completed treatment, 83.7% (384/459) in the FDC arm and 81.3% (378/465) in the LF arm (p = 0.3).

### Efficacy analysis and early response

**Fig 2:**
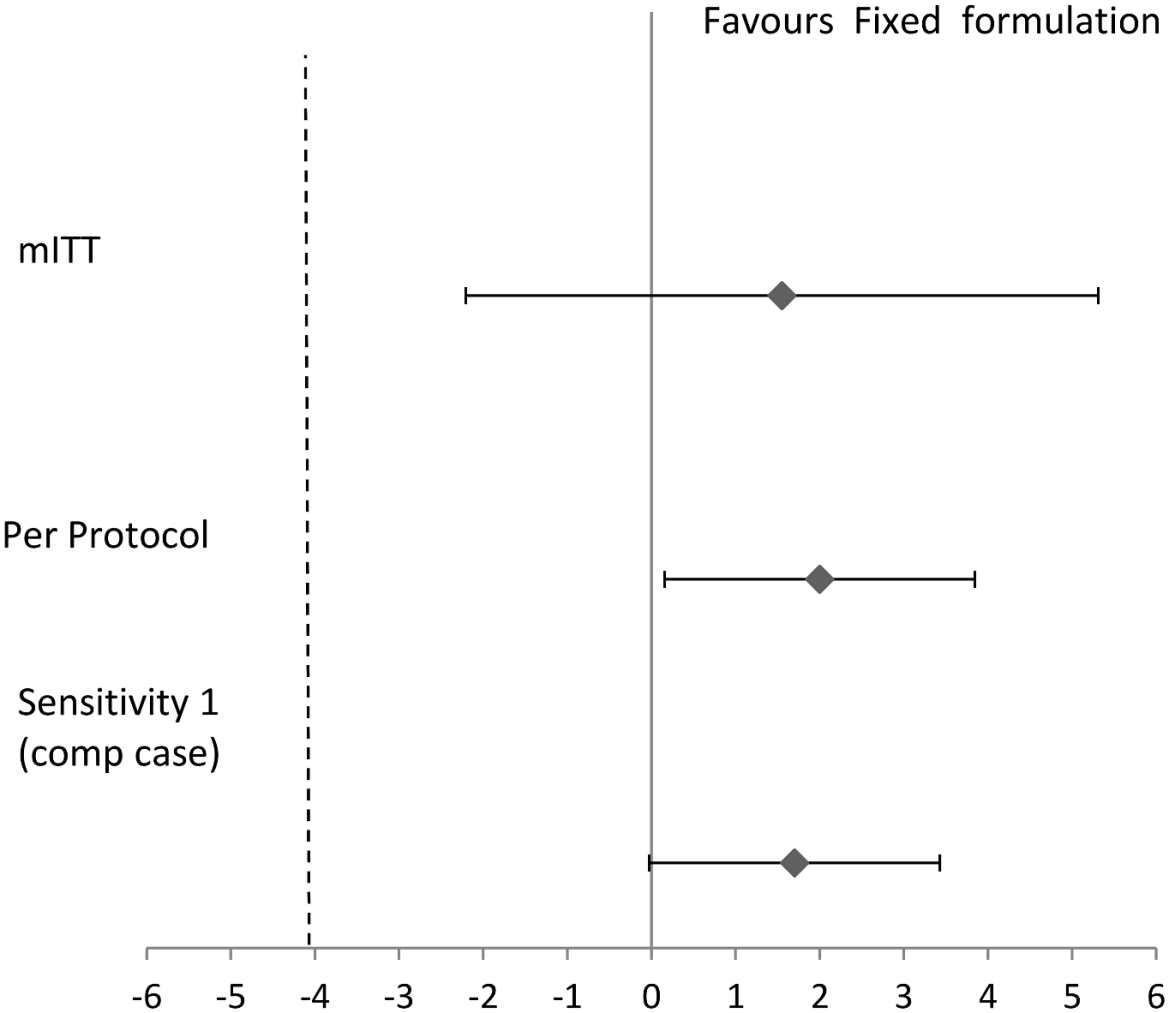
Evidence of Non Inferiority. The dotted lines indicate the margin on non-inferior for the difference between the FDC and the LF region. Error bars represent the 90% confidence interval in the risk difference. In Sensitivity 1 is a complete case analysis where all missing EOT culture results in the mITT cohort are excluded. PP indicates per protocol.

Results of efficacy analysis using mITT cohort and PP cohort are summarized in Table 2 and Fig 2. Of the FDC group, 398 participants and 396 in the LF group were culture negative at end of treatment which resulted in a cure (favourable result) of 86.7% (398/459) in FDC group and 85.2% (396/465) in LF group (difference: 1.5%; 90% CI: -2.2-% to 5.3-%). The non-statistical significance of the difference in the proportion of patients cured was also maintained when the analysis was repeated in the PP cohort. In the FDC group, 98.9% (359/363) had a favourable outcome versus 96.9% (345/356) in the LF group (difference 2.0%; 90% CI: 0.1 to 3.8). In the second analysis using the complete case scenario, 98.8% (398/403) in the FDC compared to 97.1% (396/408) in the LF group had a favourable outcome (difference of 1.7%: 90% CI: 0.0% to 3.4%). This difference also falls within the pre-specified margin of non-inferiority.

**Table 2:** mITT and Per protocol analysis at end of treatment.

	**4FDC arm**		**LF arm**			
**mITT Cohort**	N	%	N	%	**Difference**	**(90%CI)**
Unfavourable Result	61	13.3%	69	14.8%		
Favourable Result (cure)	398	86.7%	396	85.2%	1.55%	(-2.22; 5.32)
**Total**	**459**	**100.0%**	**465**	**100%**		
	**4FDC arm**		**LF arm**			
**Per Protocol Cohort**	N	%	N	%	**Difference**	**(90%CI)**
Unfavourable Result	4	1.1%	11	3.1%		
Favourable Result (cure)	359	98.9%	345	96.9%	2.0%	(0.13; 3.8)
**Total**	**363**	**100.0%**	**356**	**100.0%**		

The FDC arm had 20 HIV+ study participants, 16 of whom (80.0%) were cured of TB. Similarly, 24 (80.0%) of 30 HIV+ study participants in the LF arm were cured of TB. However, the observed difference, was not statistically significant (90% CI: -19.0% to 19.0%).

Early conversion rates in the mITT cohort were 79.5% (365/459) for the FDC group and 77.9% (362/465) for the LF group. In the PP cohort among those with month 2 culture results, early conversion rates were similar: 85.6% (303/354) in the FDC group and 83.0% (293/353) in the LF group.

### Recurrence

Based on the programmatic definition of recurrence utilizing microscopy, there were two and eleven cases of recurrence, respectively, in the FDC and LF arms in one of the 36, 48, 72 or 96 week follow-ups. Only one of these cases in the LF arm was smear positive at two time points (i.e., week 36 and week 48) and that particular patient was confirmed culture positive at week 48.

### Safety analysis

Results from safety analysis are summarized in Table 3. In both treatment arms, the occurrence of drug related adverse events as well as serious adverse events were more frequent during the intensive phase compared to the continuation phase. However, there was no significant difference in patient safety between the two treatment arms during intensive as well as continuation phases. Similarly, during the whole period of chemotherapy the occurrence of drug related adverse events (difference in proportion: -0.8%; 90% CI: -4.5%– 2.9%), serious adverse events (difference in proportion = -1.4%; 90% CI: -3.6%– 0.8%) or mortality (difference in proportion = -0.1%; 90% CI: -1.6%– 1.2%) was similar in both groups.

**Table 3:** Comparison of safety of FDC and loose formulation of anti TB treatment during the intensive and continuation phases of TB treatment.

	% (events/at risk)		
Characteristic	FDC	Loose	Difference
Serious adverse event			
Intensive phase	1.2% (6/500)	2.3% (12/500)	-1.2 (-2.8; 0.5)
Continuation phase	1.2% (6/483)	1.4% (7/478)	-0.2 (-1.8; 1.3)
Over the course of treatment	2.4% (12/500)	3.8% (19/500)	-1.4 (-3.6; 0.8)
**Death**			
Intensive phase	0.6% (3/500)	0.8% (4/500)	-0.2 (-1.4; 1.0)
Continuation phase	0.4% (2/483)	0.4% (2/278)	-0.0 (-1.0; 1.0)
Over the course of treatment	1.0% (5/500)	1.2% (6/500)	-0.1 (-1.6; 1.2)
**Drug related adverse events**			
Intensive phase	6.9% (37/500)	7.4% (40/500)	-0.5 (-3.6; 2.6)
Continuation phase	3.4% (17/483)	3.6% (18/478)	-0.2 (-2.6; 2.1)
Over the course of treatment	10.6% (59/500)	13.3% (64/500)	-0.8 (-4.5; 2.9)

## Discussion

Our trial results provide additional important data on the use of FDCs compared to LFs, in the context of actual medical practice in programmatic settings within health services in two high TB/HIV-burden African countries—Nigeria and Ethiopia. There were five main findings: (i) Non-inferiority in terms of efficacy in all three analyses (mITT, PP, and mITT_cc_), in two geographically distinct high TB/HIV endemic sub-Saharan African countries; (ii) Similar overall efficacy at the end of short course chemotherapy for TB for both FDC and LF treatment arms; (iii) Similar patient adherence to TB drug treatment for both FDC and LF; (iv) Similar drug-related adverse events in both treatment arms; and (v) HIV infection did not impede the performance of FDC and the safety profiles (even though numbers were small) were similar to non-HIV infected TB patients.

Whilst our study is similar in design and objectives to published studies [13–15] it differs by evaluating efficacy, safety and adherence to drug regimens during the entire 24-week treatment period. In Study C [13] and in Blomberg and Fourie's study [16] the intervention FDCs, were given only during the eight-week intensive phase of treatment. Comparing FDCs and LFs throughout the course of treatment (6 months) enabled us to collect information on the continuation phase of treatment—a crucial period when patients are likely to default. Furthermore, our trial is the first to demonstrate non-inferiority in terms of efficacy in all three cohorts (mITT, PP and mITT_cc_) between the two arms of the study, in two geographically distinct, high TB/HIV endemic African countries.

Our study did not observe any significant difference in adverse events between the two arms. This is consistent with findings from Study C and Blomberg and Fourie’s study [13,16] confirming that the safety of anti-TB drugs was not dependent on fixed dose formulations [13,17]. However, studies by Gravendeel et al. [18] and Su and Perng [19] found that patients on the FDC regimen had adverse events less frequently than those on the LF.

There was no difference in DOT between hospital and health-care centres. Patients who were admitted to hospital stayed only for the duration of the intensive phase (first 2 months), solely for the purpose of DOT. Thereafter they were discharged to clinic DOT. Patient adherence to FDCs and LFs was similar in both arms throughout the 24 weeks of treatment. Whilst not statistically significant, we observed a trend indicating that LF recipients were more likely to experience treatment failure. This suggests the possibility of improved performance with FDCs although this observation appears contrary to adherence levels found in other studies [8]. The reduction of pill burden may be an important factor in improved adherence and prevention of the emergence of drug resistance when FDCs are used [17,20]. However, this has not been demonstrated in this study and may require a larger study with a longer observational period to elicit this effect, if present.

Previous studies have not been able to adequately document the performance of FDCs in patients with TB/HIV co-infection treated within national programme settings [13, 21]. By design, the high HIV co-infection rates in Ethiopia and Nigeria would have allowed our study to evaluate the performance of FDCs among HIV-infected TB patients but the stringent eligibility criteria precluded enrolment of sufficient numbers. HIV-infected TB patients within the treatment thresholds of their national control programmes were excluded by the need to retain a homogeneous cohort of comparable patients. The resultant small numbers of HIV-infected TB patients prevented statistical comparison of any differences to HIV-uninfected TB patients.

The stringent enrolment criteria also may have impacted on the requisite number of patients with TB drug relapse and treatment failure. This may have contributed to our inability to evaluate the performance of FDCs in this category of patients. Analysis of the small numbers of HIV-infected TB patients did not indicate any significant difference in the cure rate. The HIV-infected TB study participants had CD4 counts above the antiretroviral treatment initiation in both countries. Some studies have elicited an alteration in the absorption of anti-TB drugs in cases of severe immune deficiency [22]. Further investigations involving a larger cohort of HIV-infected TB patients are warranted and should ascertain the efficacy and safety of FDCs in comparison to LFs; demonstrate any preferential adherence in favour of FDC or LFs among HIV-infected TB patients; and the potential for an impact on recurrences, relapse and treatment failure.

Our study had limitations which may have affected outcomes. The study subjects were recruited from the routine TB programmes, due to study inclusion and exclusion criteria, many patients with pulmonary TB who may have benefitted from the study were not enrolled in the study. Subjects were not blinded to their treatment and this could be argued as a source for potential bias due to increased safety awareness. However, the primary outcome measure was bacteriology. This was performed by laboratory technicians blinded to the treatment allocation and there was no difference in adherence between treatment arms, as also evidenced by the non-significant differences in efficacy between treatment arms.

The findings of our study are of practical relevance for medical practice within national TB programmes in Africa. Non-inferiority in efficacy and the non-significant difference in safety profiles between FDC and LF provide a further scientific evidence base for validating WHO recommendations for the use of FDCs by national TB programmes in high TB-endemic African countries. FDC appears to be as effective and safe as LF and should continue to feature in the treatment arsenal of national TB control programmes. An additional important aspect of this study was the effective regional cooperation between national TB control programmes and research institutions– ensuring programme relevance and optimal standards of study conduct and capacity development.

## Supporting Information

S1 Dataset Raw dataset used for analysis. (ZIP)

S1 Text 4FDC Protocol Final Version. (DOC)

S2 Text CONSORT 2010 Checklist 4FDC. (DOC)
