# Supplementary material for: Efficacy and Safety of ‘Fixed Dose’ versus ‘Loose’ Drug Regimens for Treatment of Pulmonary Tuberculosis in Two High TB-Burden African Countries: A Randomized Controlled Trial
Source: PLoS One. 2016 Jun 20;11(6):e0157434. doi: 10.1371/journal.pone.0157434 (PMC4913909; doi:10.1371/journal.pone.0157434)
Supplement: S1 Text — (DOC) [file pone.0157434.s002.doc]

**
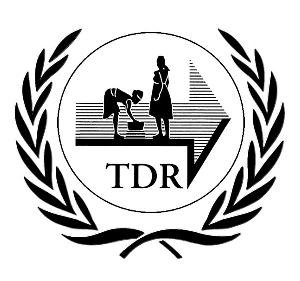
**

###### UNDP/World Bank/WHO Special Programme for Research & Training in Tropical Diseases (TDR)

TITLE: **A TWO-ARM SINGLE-BLIND RANDOMISED COMPARISON OF FOUR FIXED -DOSE COMBINATIONS VERSUS STANDARD TREATMENT WITH SEPARATE ANTI-TB DRUGS FOR TREATMENT OF PULMONARY TUBERCULOSIS**

PRODUCTS: Test arm: 4 FDC formulation (Ethambutol (275 mg)/ Rifampicin (150 mg)/ Isoniazid (75 mg)/ Pyrazinamide (400 mg) followed by 2 FDC formulation (Rifampicin (150 mg)/ Isoniazid (75 mg))

Control arm: Loose formulations of Ethambutol (400 mg), Rifampicin (450 mg), Isoniazid (225 mg), Pyrazinamide (400 mg) followed by loose formulations of Rifampicin (150 mg), Isoniazid (225 mg) and Isoniazid (100 mg)

Version: Final (July 2008)

Design: Single-blind, randomised controlled trial

Study site: Multi-centre (Ethiopia, Nigeria)

Principal Investigator: Dr. Abraham Aseffa (Ethiopia)

Dr. Joseph Chukwu (Nigeria)

Study Statistician: Dr. Lawrence Yamuah

Study Director: Dr. Philip Onyebujoh IRM/TDR/WHO

Project Manager: Dr. Mahnaz Vahedi (WHO)

TABLE OF CONTENTS

A TWO-ARM SINGLE-BLIND RANDOMISED COMPARISON OF FOUR FIXED-DOSE COMBINATIONS VERSUS STANDARD TREATMENT WITH SEPARATE ANTI-TB DRUGS FOR TREATMENT OF PULMONARY TUBERCULOSIS [1](#__RefHeading___Toc130201393)

2. RATIONALE AND OBJECTIVES [2](#__RefHeading___Toc130201394)

2.1 Rationale [2](#__RefHeading___Toc130201395)

2. 2 General Objectives [3](#__RefHeading___Toc130201396)

**Primary Outcome measures** [3](#__RefHeading___Toc130201397)

3. TRIAL STRUCTURE [4](#__RefHeading___Toc130201398)

3.1 Study Population [4](#__RefHeading___Toc130201399)

3.2 Entry Criteria [4](#__RefHeading___Toc130201400)

**Inclusion criteria** [4](#__RefHeading___Toc130201401)

**Exclusion criteria** [4](#__RefHeading___Toc130201402)

3.3 Trial Design [5](#__RefHeading___Toc130201403)

3.4 Treatment Regimens [5](#__RefHeading___Toc130201404)

**Study drugs:** [5](#__RefHeading___Toc130201405)

**Concomitant Medications:** [7](#__RefHeading___Toc130201406)

3.5 Treatment Allocation [7](#__RefHeading___Toc130201407)

3.8 Special considerations relating to patient counselling and HIV testing [9](#__RefHeading___Toc130201408)

3.9 Special considerations relating to HIV patient management [10](#__RefHeading___Toc130201409)

4. STATISTICAL CONSIDERATIONS [10](#__RefHeading___Toc130201410)

5. ASSESSMENT OF SAFETY & TOLERABILITY [10](#__RefHeading___Toc130201411)

5.2 Serious and Unexpected Adverse Events [11](#__RefHeading___Toc130201412)

6.0 DATA MANAGEMENT [11](#__RefHeading___Toc130201413)

6.1 Case Record Form (CRF) [11](#__RefHeading___Toc130201414)

6.2 Data management and quality assurance [11](#__RefHeading___Toc130201415)

6.4 Data analysis [12](#__RefHeading___Toc130201416)

7. ETHICAL AND ADMINISTRATIVE ISSUES [12](#__RefHeading___Toc130201417)

7.1 Ethics [12](#__RefHeading___Toc130201418)

7.2 Ethical Committee Approval [12](#__RefHeading___Toc130201419)

7.3 Informed consent [12](#__RefHeading___Toc130201420)

7.4 HIV testing [12](#__RefHeading___Toc130201421)

7.5 Administrative matters [13](#__RefHeading___Toc130201422)

7.6 Reports and publications [13](#__RefHeading___Toc130201423)

7.7 Protocol Amendments [13](#__RefHeading___Toc130201424)

# A TWO-ARM SINGLE-BLIND RANDOMISED COMPARISON OF FOUR FIXED-DOSE COMBINATIONS VERSUS STANDARD TREATMENT WITH SEPARATE ANTI-TB DRUGS FOR TREATMENT OF PULMONARY TUBERCULOSIS

1. BACKGROUND INFORMATION

Tuberculosis (TB) continues to be a major global public health problem **^1-5^**. Approximately two billion people worldwide are infected with *Mycobacterium tuberculosis* (MTb). The global incidence of active tuberculosis (TB) is estimated at 8.8 million new cases per year—25,000 new cases each day **^2^**. In developing countries—home to 95% of TB cases and 98% of TB deaths—tuberculosis causes 25% of the burden of preventable diseases. In 1991, the World Health Assembly adopted targets of detecting 70% of all infectious TB cases and curing at least 85% of detected cases by 2005.^2^ Despite intensified efforts, these unexceptional targets have not yet been met.^3^ At the end of 2005, 82% of TB cases were successfully treated, but only 45% of infectious TB cases were detected.^4^ Detection and cure rates are especially low in sub-Saharan Africa and other high HIV prevalence settings.^5^

Combined preparations of antituberculosis drugs have been widely used. The use of fixed-dose combined (FDC) drugs in the treatment of tuberculosis by National Tuberculosis Programmes (NTPs) has been recommended by the World Health Organisation (WHO) and the International Union Against Tuberculosis and Lung Disease (IUATLD).^6^ There may be many advantages of FDC drugs; they could aid compliance and hence increase cure rates, could include preventing the emergence of drug resistance due to monotherapy, reduce the risk of incorrect dosage and simplify procurement and prescribing practices.

The recommended treatments for newly diagnosed tuberculosis always contain an initial intensive phase of four drugs. The most widely used four-drug regimen consists of rifampicin, isoniazid, pyrazinamide and ethambutol. Two and three-drug FDCs, containing isoniazid and rifampicin, have been in use for a number of years. Several pharmaceutical companies have manufactured a combined fixed dose tablet containing these four drugs. Their bioavailability levels have been evaluated as acceptable when compared to those given in separate drug formulations.^7,8^ It has been reported that the combined tablets are well tolerated and the rate of side effects similar to that of the separate formulations. ^9, 10^

# 2. RATIONALE AND OBJECTIVES

# 2.1 Rationale

Owing to the length (6-8 months) and complexity (4 drugs, up to 16 tablets a day) of the current treatment, the delivery of short-course chemotherapy for TB with single drug formulations is challenging and poses risks of prescription errors and lack of compliance with treatment where DOTS is either poorly or not used. Drug supply management is complicated, since individual drugs must frequently be ordered from different sources, and there is a risk of out-of-stock situations, in which patients may be treated with too few drugs, or even only one drug in isolation. Another major concern in the treatment of tuberculosis is to prevent the emergence of drug resistance especially to isoniazid and rifampicin, the two most potent antitubercular drugs.

One approach to tackling these challenges is to use fixed dose combinations (FDCs) of the four and two TB drugs that are used in the intensive and maintenance phases, respectively. However, these drugs have been little used to date despite being recommended by the WHO and the IUATLD. One concern is the variable bioavailability of rifampicin in poor quality fixed dose combinations. Some rifampicin containing FDCs with adequate absorption in healthy normal volunteers have been endorsed by the WHO.^6^Poor absorption may occur in HIV infected individuals.

The advantages of FDCs over loose drugs could include the following:

- The emergence of HIV/AIDS has complicated treatment of TB among co- infected patients (HIV-infected TB cases). The increasing availability of HAART (highly active antiretroviral therapy) in the public sector in resource-limited settings and the increasing mortality among HIV-infected TB cases managed alone with TB drugs implies the need for concomitant treatment of HIV and TB. 4FDCs presents a rational approach to managing the huge burden that would otherwise ensue in these categories of patients, which could lead to drug resistance from dropping off some TB medications in loose formulations.
- It is expected that 4 FDCs limit the risk of emergence of drug resistant tuberculosis by using a simplified treatment regimen and increasing treatment compliance which may reduce the probability of monotherapy^11^. In a study conducted in Hong Kong comparing 3FDCs (Isoniazid, Rifampicin, Pyrazinamid) with loose anti TB treatment, it is reported that only 1% of 312 patients who received 3FDCs complained about difficulty of swallowing or the quantity of drugs to be ingested compared with 5% of 308 patients receiving the loose preparations. Although it is expected that 4FDCs may therefore improve the patient's compliance with treatment, to date no double blind randomized trials have been conducted to evaluate the patient's compliance with 4FDCs^12^ .
- With single drug formulations, there are at least three reasons which contribute to the drug non-availability: inadequate stock, delay in receipt of orders and no replacements available on reaching the expiry date. The above issues could also exist with the 4FDCs treatment, however because of fewer drug formulation orders, shipments and distribution involved, the efficacy of drug supply system could be well improved^11^.
- As rifampicin is also used for treatment of some other common infectious diseases such as atypical pneumonia and sexually transmitted diseases, black-market sales of this drug are not uncommon in countries where other antibiotics are unavailable^11^. Therefore, use of rifampicin as a broad spectrum antibiotic for conditions other than tuberculosis could lead to its usage as mono-therapy to patients who may also suffer from tuberculosis. Treating a tuberculosis patient with mono-therapy of rifampicin rapidly leads to resistance even if it is given for a short period only. In combination drugs, the presence of isoniazid could reduce the probability for the survival of rifampicin-resistant mutants.
- Due to the enormous costs involved in treating drug-resistant tuberculosis, preventing the emergence of drug resistance must be given the highest priority. Mitchison argues that multiple interruptions of treatment regimes are considered the predominant cause of drug resistance TB^13^. When using single-drug formulations, due to the length (6-8 months) and complexity (four drugs, up to 16 tablets per day) of the treatment patients are more prone to continue their treatment with one drug while interrupting the others, thus it creates a risk of mono-therapy and selection of drug resistant mutants. Hence a clinical trial should be conducted to address such issues.
- In the past, some researchers claimed that adverse reactions to 3FDCs are not more common than loose combination anti-TB. To date, no data is available on the safety and adverse reactions associated with 4FDCs.

In conclusion, if their potential is realized, the 4FDCs and 2FDCs can be a means of improving TB control but FDCs have not been assessed adequately in countries like Ethiopia and Nigeria where HIV seropositivity among TB patients is high. Therefore, there is a need to increase the evidence base on which to recommend FDCs in TB control programme and other settings.

## 2. 2 General Objectives

To measure the efficacy and assess safety of fixed dose combination therapy (FDCs) compared to separate loose formulation therapy in pulmonary tuberculosis.

###

### ***Primary Outcome measures***

- cure rates at end of treatment (6 months). A cure is defined as one negative sputum culture in patients who did not fail treatment. A treatment failure is defined as:

1. a patient who is persistently smear positive by five months of treatment
2. a patient who becomes smear negative but positive again at or after five months of treatment (note: sputum positivity before this time is not considered a treatment failure)

- ***Secondary outcome measures***
- early response rates: proportion of patients with negative culture results at 8 weeks after initiation of therapy
- proportion of patients with relapses during the 72 weeks follow up period after the end of treatment. A relapse is a patient previously treated for tuberculosis who has met the case definition of cure (as defined above) and been declared cured by a physician and who has now been diagnosed with bacteriologically positive (two positive smears and culture positive) tuberculosis. If there is discordance between smear and culture results, a clinical decision will be made regarding further management: (i) smear positive, culture negative, and (ii) smear negative and culture positive.
- cure rates in HIV positive TB patients
- proportion of patients with clinical deterioration of pulmonary tuberculosis with the need for hospitalisation
- proportion of patients with *serious* adverse events any time during chemotherapy
- proportion of patients with any adverse events during chemotherapy
- proportion of patients completing treatment (defined as patients who achieved a total of 54 out of 56 doses in the first eight weeks of treatment AND 107 out of 112 doses at a daily rate during the subsequent 16 weeks of treatment) within each group. (96% doses taken considered as completed in intensive and maintenance phases).
- Proportion of patients who developed MDR-TB

# 3. TRIAL STRUCTURE

## 3.1 Study Population

Patients presenting to a TB clinic clinically suspected pulmonary TB.

## 3.2 Entry Criteria

### ***Inclusion criteria***

Patients with newly diagnosed pulmonary tuberculosis (with or without pleural effusion) will be admitted to the study provided they fulfil the following criteria:

- two sputum specimens positive for tubercle bacilli on direct smear microscopy. A patient is considered smear positive if: (i) 2 out of 3 consecutive sputum smears are read as positive, or (ii) 2 out of 5 are read as positive in the event that only one of the first three smears is positive, in which case the patient will be asked for two more specimens: one morning and one spot.
- no history of previous anti-tuberculosis chemotherapy, or antiretroviral therapy.
- aged 18 years and over
- a firm home address and intent to remain there during the entire treatment and follow up period
- informed consent to participate in the study.
- weight between 40.00 kg and 70.00 kg inclusive.
- acceptance of HIV counselling and testing
- CD4 ≥ 220 cells/microlitre blood if the patient is HIV positive in Nigeria
- CD4 > 350cells/microlitre blood if the patient is HIV positive In Ethiopia

### ***Exclusion criteria***

- Positive urinary pregnancy test or obviously pregnant on physical examination
- additional extrapulmonary TB (e.g. bone, meningeal, peritoneal or miliary)
- contraindications to any medications in the study regimens (e.g. documented allergy; optic neuritis, blurred vision, red-green colour blindness and poor visual acuity ; any active hepatic diseases.
- evidence (laboratory and/or clinical history) of pre-existing non-tuberculous disease likely to affect the response to, or assessment of, treatment: (i) diabetes mellitus, (ii) liver impairment, (iii) renal impairment, (iv) peripheral neuropathy, (v) terminal illness such as with malignancy.
- requirement for hospitalisation for any reason other than directly observed treatment (DOT)
- concomitant immunosuppressive treatment during the whole study period.
- psychiatric illness, alcohol or drug abuse likely to lead to uncooperative behaviour
- patients on antiretroviral treatment during TB treatment period.

***Study withdrawals***

- AFB smear positive patients found later to be culture negative. These patients will be withdrawn from the study but they will be assessed clinically to see if treatment should be continued or not. (This occurs in 1-5 % of newly diagnosed cases).
- Patients withdrawing consent in the course of the trial
- Antiretroviral therapy initiation within the first six months of anti-TB medication.
- AFB smear positive patients at week 20 whose sputum culture and DST confirm resistant to isoniazid and rifampicin (MDR TB)
- Drug toxicity necessitating interruption of treatment

Patients who withdraw from study or are lost to follow up will however still be included in the intention to treat (ITT) analyses set.

## 3.3 Trial Design

This is a two-arm single-blinded, randomised comparison of 4FDCs (intervention group) versus standard treatment with separate TB drugs (comparator group).

The investigators and laboratory personnel and those responsible for data analysis will be blinded to the allocated treatment regimen. Blinding will occur by separating treatment dispensation from clinical management and from the compilation of laboratory results. Investigators will not be blinded to HIV status as this would be essential for adequate patient management.

## 3.4 Treatment Regimens

### ***Study drugs:***

The drug regimens (see tables) will consist of 8 weeks of daily directly observed treatment (DOT) treatment, followed by 16 weeks of daily directly observed treatment. There will be two arms: (i) the intervention group - treatment will consist of 4FDCs (8weeks, i.e. **56 doses**) and 2FDCs (16 weeks, i.e. **112 doses**), and (ii) the comparator group - treatment with the same four and two drugs administered as loose formulations. The 4FDCs and the 2FDCs used will be identical across the different study sites, Nigeria and Ethiopia. Both 4FDCs and 2FDC preparations will include rifampicin. Full details of the drug regimen, including drug dosages, for each patient and of the procedure to be followed are given on the front and back of each Treatment Card.

The doses of drugs to be given to each patient are shown below and are based on the weight of the patient at the time of starting treatment.

*2. Weight between 40-54 kg (inclusive)*

*Intensive phase*.

For the patients allocated to the intervention regimen (FDCs) the dose for the first eight weeks of treatment is shown in the table below:

| MEDICATION  Combined tablet (COMB) of: | WEIGHT IN KILOGRAMMES |
| --- | --- |
|  | 40-54 |
| Ethambutol (275mg)  Rifampicin (150mg) Isoniazid (75mg) and  Pyrazinamide(400mg) | 3 tablets |

For patients allocated to the comparator regimen, the dose for the first eight weeks of treatment is shown in the table below:

| MEDICATION | WEIGHT IN KILOGRAMMES |
| --- | --- |
| as loose tablets (SEPA) | 40-54 |
| Ethambutol (400mg)  Rifampicin (450 mg)  Isoniazid (225mg)  Pyrazinamide (400 mg) | 2 tablets  1 tablet  1 tablet  3 tablets |

*Maintenance phase*.

For the maintenance phase, drugs will be administered under daily observation. The doses shown in the table below are based on the weight of the patient upon completion of the initial intensive phase:

| MEDICATION  Combined tablet (COMB) of: | WEIGHT IN KILOGRAMMES |
| --- | --- |
|  | 40-54 |
| Rifampicin (150mg)/Isoniazid (75mg) | 3 tablets |

For the comparator group, the regimen is shown below:

| MEDICATION  as loose tablets (SEPA) | WEIGHT IN KILOGRAMMES |
| --- | --- |
|  | 40-54 |
| Rifampicin (150 mg)  Isoniazid (225 mg) | 3 tablets  1 tablet |

*2. Weight between 55-70 kg (inclusive)*

*Intensive phase*.

For the patients allocated to the intervention regimen (FDCs) the dose for the first eight weeks of treatment is shown in the table below:

| MEDICATION  Combined tablet (COMB) of: | WEIGHT IN KILOGRAMMES |
| --- | --- |
|  | 55-70 |
| Ethambutol (275mg)  Rifampicin (150mg) Isoniazid (75mg) and  Pyrazinamide(400mg) | 4 tablets |

For patients allocated to the comparator regimen, the dose for the first eight weeks of treatment is shown in the table below:

| MEDICATION | WEIGHT IN KILOGRAMMES |
| --- | --- |
| as loose tablets (SEPA) | 55-70 |
| Ethambutol (400mg)  Rifampicin (450 mg)  Rifampicin (150 mg)  Isoniazid (100 mg)  Pyrazinamide (400 mg) | 3 tablets  1 tablet  1 tablet  3 tablets  4 tablets |

*Maintenance phase*.

For the maintenance phase, drugs will be administered under daily observation. The doses shown in the table below are based on the weight of the patient upon completion of the initial intensive phase:

| MEDICATION  Combined tablet (COMB) of: | WEIGHT IN KILOGRAMMES |
| --- | --- |
|  | 55-70 |
| Rifampicin (150mg)/Isoniazid (75mg) | 4 tablets |

For the comparator group, the regimen is shown below:

| MEDICATION  as loose tablets (SEPA) | WEIGHT IN KILOGRAMMES |
| --- | --- |
|  | 55-70 |
| Rifampicin (150 mg)  Isoniazid (100 mg) | 4 tablet  3 tablet |

WHO will ensure that the drug preparations are all from the same source. The manufacturer of the drugs is Lupin Pharmaceuticals**.** Bioavailability data on the RH and 4 FDC (RHZE) formulations manufactured by Lupin will be available to the study team through TDR. This includes sufficient information concerning the quality of the product, bioavailability, dissolution testing etc. to pass review by the national regulatory agency for permission to carry out the investigations.

### ***Concomitant Medications:***

Although there will be no restrictions of ancillary treatments, the names, doses preparations, indications and duration of use of such medications will be properly documented. Concomitant treatment with antiretroviral will be a reason for exclusion/withdrawal from the study.

## 3.5 Treatment Allocation

Patients are to be allocated to the different treatment arms according to a computer generated randomisation list. The randomisation list will be prepared and kept by the Study Director. Each centre will be provided with a batch of sealed envelopes. The total number of allocated envelopes will correspond to their expected intake to the study. Therefore, each patient will be allocated randomly **either** the FDCs regimen **or** the separate drug regimen. Each sealed envelope will contain a sequential patient code (randomisation number) and a Treatment Card printed with the details of one of the two regimens to which the patient will be allocated. Only a specially assigned person will open the envelope and dispense the drugs to the patient. No other study personnel will be present at the time of drug allocation. This information will, therefore, only be known to the assigned dispenser and the patient. Randomisation is considered to have taken place once the envelope containing the code for the drug regimen is opened. Once a patient is allocated a randomisation number must not be used for other patients, in the event that the patient decides to withdraw voluntary consent before study drug administration. A treatment allocation log will be kept by the specially assigned person and will not be available to the investigators until the analysis has been concluded, or unless the patient’s physician requires this information for safety reasons.

The PI and the clinicians will remain blinded to the drug regimens of individual patients throughout the study.

**3.6 Study conduct**

The flow of patients is illustrated in the flow chart (Annex ). Patients will be seen by the clinic physicians as per the clinic routine. From this consultation, any smear positive cases of pulmonary TB will be referred to the research team to assess eligibility. Consenting patients will undergo full assessment as detailed in the CRF. This will include: (i) sputum culture, (ii) HIV counselling and testing (including CD4 count), (iii) a full blood count, (iv) liver function tests (SGOT, SGPT, alkaline phosphatase, direct and total bilirubin, albumin), fasting blood sugar (v) renal function tests (creatinine, blood urea nitrogen), and (vi) a urinary pregnancy test, uric acid and urinary glucose, protein, bilirubin and urobilinogen.

Sputum specimens for direct smear will be collected on Day 0, Weeks 8, 20, and 24 (See table). At each of these times three sputum specimens will be provided: after deep cough: one spot, one morning and a second spot specimen. A patient is considered smear negative if: (i) all 3 consecutive sputum smears are read as negative, or (ii) 4 out of 5 are read as negative in the event that only two of the first three smears was negative. (Note: with TB co-infected HIV patients, more patients are smear negative).

Culture will be done on Day 0 and weeks 8 and 24. A culture and sensitivity at week 20 will only be done if sputum samples of the same week are positive. Sensitivity test on the stored sputum samples from week 0 will also be done if sputum samples at week 20 are positive. A minimum of three sputum samples will be required from each patient at each of these time points. Sample collection will be done on days when patients come to collect their drugs. Cultures will be inoculated from pooled sputum samples.

For the purpose of defining the cure and relapse rates, only the sputum culture result will be used. Standard operating procedures have been prepared for collection, storage, transport, and testing of culture samples before the study starts.

Time frame for the duration of the study:

**Enrolment** **Week 8 Week 20 Week 24**

Time zero (End of intensive phase) (End of continuation phase)

(follow up period starts )

**Week 36 Week 48 Week72 Week 96**

(End of trial)

| Study procedures | Screening | Enrolment  Study Drug  T=0h | Week 8  (end of intensive phase) | Week  20 | Week 24  (end of continuation phase) | (Weeks 36,48,72 and 96) |
| --- | --- | --- | --- | --- | --- | --- |
| Medical History | X |  |  |  |  |  |
| Physical Examination | X |  | X | X | X | X |
| Sputum for AFB smear | X |  | X | X | X | X |
| Sputum for culture |  | X | X | X^a^ | X | X^a^ |
| Sensitivity testing |  |  |  | X^b^ |  |  |
| Informed consent | X |  |  |  |  |  |
| Haematology and Clinical chemistry | X | X | X | X | X | X |
| Urine analysis | X | X |  |  |  |  |
| Pregnancy test | X |  |  |  |  |  |
| HIV testing  CD4 test | X  X^d^ |  |  |  | X^d^ | X^d^ |
| Randomisation |  | X |  |  |  |  |
| Drug administration |  | X | X | X | X^e^ |  |
| Adverse Events |  | Symptom checklist | X | X | X | X |

X^a^ It the sputum smear is positive

X^b^ Sensitivity testing of screening smear and 5 month smear if sputum is positive.

X^d^ CD4 testing only for HIV positive TB patients. Repeat CD4 test on weeks 24, 48, 72 and 96. Not indicated at week 36.

X^e^ By the end of week 24, treatment should be completed.

Patients will be instructed to attend the TB treatment centre, for the duration of treatment; both the intensive and maintenance phases. All drug doses will be administered under observation and documented by the trial nurse. All drug doses in the continuation phase will be administered under standard operation of DOTS of the country. Patients will also be evaluated for clinical, haematological and biochemical parameters during these follow up period.

Trial subjects will be followed up for 72 weeks after the completion of treatment. They will be required to come to TB treatment centre for clinical, haematological and biochemical assessments at the end of 36 weeks ,48 weeks ,72 weeks and 96 weeks after initiation of treatment. A home visitor will trace individuals who fail to come at the specified time points. At each follow-up time, subjects will provide three sputum samples: one spot, one morning and a second spot sputum specimens for smear examination at each of the three follow up visits. Cultures will be done only for smear positive individuals (as defined above).

## 3.8 Special considerations relating to patient counselling and HIV testing

All eligible patients will be informed about the relationship between HIV-infection and TB by the examining physician. The advantages of testing and knowledge about status to the patient and family will be explained. Health education will be given to the patient on the need to prevent and treat opportunistic infections, if HIV co-infected. Patients who agree to consider testing will be advised to visit a trained HIV-counsellor in the same institution. The nurse will discuss the pros and cons of testing with the patient. The patient will be encouraged to ask questions including on the confidentiality of the procedures of testing. Those patients who agree to be tested will be provided with a request form for the laboratory. The patient will meet and discuss the result with the Counsellor who will continue advising the subject over the next months as required. HIV-negative subjects will also be encouraged to have appointments with the counsellor to discuss safe practices. The counsellor will be responsible for providing information to the HIV-positive subject on available support for sero-positive individuals. The Counsellor will keep confidential written records of the discussions.

## 3.9 Special considerations relating to HIV patient management

In the interests of good patient management, HIV testing will be done as part of pre-study assessment. HIV positive patients have higher rates of recurrent disease compared to HIV negative patients. Pre- and post counselling is done routinely under national guidelines at the TB centre. Subjects found to be HIV-positive will be referred to the physician responsible for HIV-related care at the site and those with CD4 count below 220 in Nigeria and those with CD4 below 351 in Ethiopia will be excluded from this study. All enrolled HIV positive patients (i.e. patients with CD4≥220 in Nigeria and CD4>350 in ethiopia) will have follow up CD4 test at weeks 24, 48, 72 and 96. Those patients with CD4 below 200 in Nigeria and CD4 below 351 in Ethiopia during the follow up period will be referred to the physician responsible for HIV-related care including ART. Treatment of HIV positive patients in Ethiopia and Nigeria is as follows:

If anti-retroviral therapy is indicated, patients will be referred for treatment so that they can benefit from the highest possible local standard of care for HIV infection. Prophylaxis with co-trimoxazole may be recommended by the patient's physician and any medications prescribed will be documented on the CRF.

Nigeria apply the WHO guidelines for scaling up antiretroviral access in resource-limited settings, ie antiretroviral treatment should be initiated in TB patients if there is a very high risk of HIV disease progression and mortality. These would occur mainly among TB patients with CD4 T cell count <200 cells/mm^3^. However, in Ethiopia HIV infected patients with CD4 T cell count<351 are initiated on antiretroviral treatment (Guidelines for management of opportunistic infections and Anti retroviral treatment in adolescents and adults in Ethiopia, federal HIV/AIDS prevention and control office, FMOH, July 2007).

Those patients who are eligible for ART will be referred to the St Peter's Hospital and Bole Health center in Ethiopia and Ebonyi State University Teaching Hospital and ABA South LGA Hospital in Nigeria. Both hospitals are collaborating centres in the trial. Those patients who are eligible for ART-( excluded from this study) will be referred and treated at centres where ART treatment is provided based on the National guidelines in both countries.

# 4. STATISTICAL CONSIDERATIONS

The objective of the study is to demonstrate that FDCs have equivalent efficacy to the loose formulations.

Sample sizes of 498 from the intervention group and 498 from the standard group achieve 90%

power at a 5% significance level using a one-sided equivalence test of proportions when the

proportion of patients cured in the comparator group (Ps) is 96% and the maximum allowable difference (clinically acceptable difference) between proportions in the two treatment groups that still results in equivalence (the range of equivalence) is 4%. A sample size of 498 in each group is estimated with dropout rates of 10%. Two study sites will conduct the trial and contribute to the total sample size. Ethiopia will contribute a total of 498 subjects (249 in each arm) per country to reach the sample size of 996.

# 5. ASSESSMENT OF SAFETY & TOLERABILITY

**5.1 ADVERSE EVENTS**

Safety will be assessed by monitoring the occurrence of adverse events. An adverse event is a symptom, sign, clinical illness, or an abnormal laboratory result that was either: (i) not present on Day 0 but occurred during follow-up, or (ii) was present on Day 0 but has since become worse. There will be a checklist of symptoms and signs that will be sought at each assessment (see CRF). Routine laboratory tests (detailed in the CRF) will be done at baseline and repeated only as clinically indicated during follow-up. Other laboratory tests will done if clinically indicated.

**All adverse events will be graded as: 0=normal, 1=mild, 2=moderate, 3=severe** according to ICH Clinical Safety Data Reporting. An assessment of the cause of the adverse event will be done by the clinicians. The relationship to study drug will be graded as (i) none, (ii) unlikely, (iii) possible, (iv) probable, (v) definite, (vi) unknown. Refer to the WHO/TDR SOP for these definitions. Adverse events will be treated on their merits and patients followed up as necessary. All adverse events will be recorded in the CRF. All serious and unexpected adverse events, as defined in the SOPs, will be recorded in the SAE form (see below). Necessary details will include: (i) the description of adverse event, (ii) onset date, (iii) speed of inset: sudden, gradual, unknown, (iii) duration, (iv) severity, (v) relationship to study drugs, (vi) outcome, (vii) measures taken (symptomatic treatment, discontinuation of treatment).

## 5.2 Serious and Unexpected Adverse Events

A serious adverse event is defined as: (i) fatal, (ii) life threatening, (iii) requires acute medical treatment, (iv) permanently disabling, (iv) required hospitalisation for treatment, (v) prolongs a hospital stay, (vi) causes malignancy, (vii) causes a congenital abnormality. An unexpected adverse event is defined as an adverse event, the nature or severity of which is not consistent with the applicable product information (Package Insert or Summary of Product Characteristics).

All SAEs will be reported immediately or not later than 24 hours by telephone, facsimile, or email to (Dr. Mahnaz Vahedi or delegated person) at WHO/TDR. The telephone report should be followed by a written report that should fully document the event. Additional laboratory information and clinical data should be supplied as soon as possible, not exceeding 5 days following the event. In case of death, if an autopsy is performed, a copy of the pathological report should be sent to the Sponsor. When the SAE has resolved, a detailed report will be submitted to TDR with a copy kept in the investigator's file.

#

# 6.0 DATA MANAGEMENT

## 6.1 Case Record Form (CRF)

Patient information will be collected in the study Case Record Form (Appendix 1). The CRF must be completed legibly for each patient enrolled in the study, in black ink and signed by the Principal Investigator or authorised trial personnel. Patient records will be kept such that the identity of the patient will not be disclosed, and will be available for inspection by the clinical monitor and the Study Director. For identification purposes, there will be three or four unique identifiers on each form and specimen: initials (name, father's name, grandfather’s name; there are no surnames in Ethiopia), randomisation number, clinic number, and date of sample.

## 6.2 Data management and quality assurance

Sputum specimens will be subject to blind rereading. A SOP for the laboratory will be completed before the trial is initiated. Laboratory equipment will be calibrated according to the manufactures' recommendations (and outlined in the SOP). TDR/WHO as the sponsoring agency will perform monitoring visits and subject data recorded in the CRFs to source data verification.

## 6.4 Data analysis

The database file generated thus will be converted into statistical package STATA software version 7 (STATA Corporation, Texas, USA) and analysed by the study statistician, and PI in collaboration with the study director. Analysis will be both (a) by intention-to-treat, and (b) per protocol (excluding those who did not receive adequate chemotherapy or were withdrawn from the study), including survival analysis. Subjects will be compared based on their assigned treatment using chi-square, Student's t-test or the Wilcoxon rank sum test as appropriate depending on the scale of measurement and the validity of underlying assumptions for the baseline characteristics. Primary and secondary outcomes for each study subject will be recorded as positive (desirable) and negative (not desirable) at a pre-specified time. For primary outcome Chi-square will be used to compare proportions of culture positive and culture negative subjects at the end of the study in the two treatment groups. The effects of confounding variables (such as age and sex) will be adjusted using multiple logistic regression. In model construction, backward elimination will be employed using important independent variables initially. An alpha of 0.10 will set the significance level for entering a variable into the model. Chi-square will also be used to compare differences in each of the three secondary end points. In the whole analysis, an alpha of 0.05 will set the significance level for testing the null hypothesis. In presenting results exact p-value as obtained from the computer program will be presented.

**6.5 Analytical plan**

The analytical plan will be finalised before initiation of the analytical phase of the study.

# 7. ETHICAL AND ADMINISTRATIVE ISSUES

## 7.1 Ethics

This clinical study will be conducted in accordance with the principles laid down by the National Guidelines and the World Health Assembly of 1975 on Ethics in Human Experimentation and the Helsinki Declaration. This study will adhere to the standards established for Good Clinical Practices and conform to the TDR Standard Operating Procedures.

## 7.2 Ethical Committee Approval

Written approval to perform this trial will be obtained from the local Institution Review Board (AHRI/ALERT Ethical Clearance Committee), the Ethiopian and Nigerian National Ethical Clearance Committee and the WHO’s ERC , Geneva, Switzerland.

This amendment will be submitted and approval will be obtained from the Ethiopian and Nigerian National Ethical Clearance Committee and the WHO’s ERC, Geneva, Switzerland.

## 7.3 Informed consent

Each subject will receive detailed information (see informed consent form) and possible risks of the trial. This also includes information on HIV testing and counselling. Written informed consent will be obtained from every subject participating in the study. The subject will be at liberty to abstain from participation in the study and to withdraw consent at any time without compromising the regular treatment the subject is entitled to for his/her clinical condition.

## 7.4 HIV testing

All patients will be informed that they have the right to refuse HIV testing when it is offered. HIV testing will be mandatory for entry into the study. All patients who agree to HIV testing will receive pre- and post test counselling. The investigators will make every effort to offer voluntary testing and counselling and HIV testing to study participants so that VCT uptake is high. In addition, a serious attempt will be made to link subjects who are HIV positive to services that provide antiretroviral treatment where indicated.

## 7.5 Administrative matters

The Principal Investigator will prepare and maintain a file containing the essential documents for the conduct of a clinical trial as listed in the ICH Guidelines for Good Clinical Practices.

Patient records will be kept such that the identity of the patients will not be disclosed, and will be available for inspection by the clinical monitor and Study Director. CRFs of each study subject will be archived for 3 years. Arrangements will be made by the Sponsor so as to archive them for a longer period, as necessary.

Direct access to source data/documents relating to the trial should be granted to the sponsor for trial-related monitoring, audits, Ethical Committee review and regulatory inspections.

## 7.6 Reports and publications

The primary report of the study will be analysed by the WHO/TDR. It is agreed that publication of this study will be permitted, authorship to include all experts who have contributed to the development of this study, its analysis and interpretation. It is understood that there will be, prior to the submission of the manuscript, agreement on the data and their interpretation with the Study Director.

The Study Director/WHO will be free to use the data for the development of TB policy, on the understanding that on any such occasion there will be full and proper attribution to the source of the material and results. Such development or deployment may happen under the sole control of the WHO or in conjunction with the authorised partners of the WHO for the purpose of such development and/or deployment. The permission of the authors of the material will not need to be specially sought for such development or deployment. WHO will own the data but this will not in any way prevent the PI s from publishing the work.

## 7.7 Protocol Amendments

After the protocol has been signed, no changes may be made without the agreement of the PI, the Study Director and Sponsor. Any change will be signed and dated by all parties, attached to the original protocol and submitted for Ethical Committee review.

**Flow chart for MDR TB**

Week Zero

Intensive phase 2(HREZ)

Repeat sputum microscopy at

end of week eight

**If sputum smear negative** **If sputum smear positive**

Switch to continuation phase Continue with the intensive phase regimen until week12

Continuation phase (4HR daily regimen) Continuation phase (4HR daily regimen)

From end of week 8 to end of week 24 From end of week 12 to end of week 24

Repeat sputum smear microscopy

at week 20

If sputum smear negative If sputum smear positive

Send for culture and DST

Continue with 4HR until end week

24 **Exclude** from the study if Resistant to Isoniazid and Rifampicin-

For MDR-TB, refer to greenlight institution

**Recruitment flow chart**

**Suspected TB patient**

**Smear positive (2/3 or 2/5)**

**History/examination**

**HIV testing and counselling**

**Exclude if indicated**

**Inclusion and exclusion criteria**

**Exclude if indicated**

**Consent for trial entry**

**Further blood tests:**

**(FBC, EUC, LFT, Fasting BSL,**

**Urinary Pregnancy Test)**

**CD4 count if indicated**

**Exclude if indicated**

**Enrolment**

**Treatment allocation**

**Table 1. Recommendations for individuals with TB disease and HIV co-infection**

| CD4 cell count | Recommended regimen | Comments |
| --- | --- | --- |
| CD4 <200mm3 | Start TB treatment. Start ART as soon as TB treatment is tolerated (between 2 weeks and 2 months) ^a^  EFV-containing regimens^b, c, d^. | Recommend ART.  - EFV is contraindicated in pregnant women or women of childbearing potential without effective contraception. |
| CD4 200−350/mm3 | **Start TB treatment.**  Start one of the regimens below after the initiation phase (start earlier if severely compromised):  EFV-containing regimens ^b^  or NVP-containing regimens in case of rifampicin-free continuation phase TB treatment regimen. | **Consider ART.**  **Start ART after 8 weeks( after intensive phase) of TB treatment** |
| CD4 >350mm3 | Start TB treatment. | Defer ART^e^ |
| CD4 not available | Start TB treatment. | Consider ART ^a, f^ |

Adapted from WHO/HTM/HIV2004 - Scaling up antiretroviral therapy in resource-limited settings: Treatment guidelines for a public health approach - **2003** revision.

^a^ Timing of ART initiation should be based on clinical judgement in relation to other signs of immunodeficiency. For extrapulmonary TB, ARVs should be started as soon as TB treatment is tolerated, irrespective of CD4 cell count.

^b^ Alternatives to the EFV portion of the regimen include: Saquinavir/Ritonavir (SQV/r) (400/400 mg bid), SQV/r (1600/200 mg qd in sgc), Lopinavir/ Ritonavir (LPV/RTV) (400/400 mg bid) and Abacavir (ABC).

^c^ Nevirapine (NVP) (200 mg qd for two weeks followed by 200 mg bid) may be used in place of Efavirenz (EFV) in absence of other options. NVP-containing regimens include: Stavudine/Lamivudine/ Nevirapine (d4T/3TC/NVP) or Zidovudine/Lamivudine/Nevirapine (ZDV/3TC/NVP).

^d^ EFV-containing regimens include d4T/3TC/EFV and ZDV/3TC/EFV.

^e^ Unless non-TB Stage IV conditions are present; otherwise start ARVs upon completion of TB treatment.

^f^ If no other signs of immunodeficiency are present and patient is improving on TB treatment, ARVs should be started upon completion of TB treatment.

**Table 2. Guide for the management of patients presenting with TB before initiation of ART**

Guidelines for management of opportunistic infections and Anti retroviral treatment in adolescents and adults in Ethiopia, federal HIV/AIDS prevention and control office, FMOH, July 2007).

|  | **Recommendation** | **Preferred ARV regimen** |
| --- | --- | --- |
| CD4 count <200/mm^3^ | - Start TB treatment. - Start ART as soon as TB treatment is tolerated (usually between 2 -8 weeks of TB treatment)^1^ | EFV containing regimen is preferred.^2^ However, if drugs are not available or there are problems with EFV (adverse effects with intolerance and risk of pregnancy) NVP containing regimen may be given with caution, monitoring ALT every month (3). |
| CD4 count 200-350/mm^3^ | - Start TB treatment. - Start ART after 8 weeks (after intensive phase) of TB treatment | - Start NVP containing regimen if the continuation phase does not include Rifampicin. - Start EFV containing regimen if the continuation phase includes Rifampicin. |
| CD4 > 350/mm^3^ | - Start TB treatment - Defer ART | - Re-assess eligibility for ART at 8 weeks, in the course of TB-treatment, at completion of TB treatment, or as indicated. |
| CD4 not available | - Start TB treatment. - Start ART after 2-8 weeks of TB treatment | - Start NVP containing regimen if the continuation phase does not include Rifampicin. - Start EFV containing regimen if the continuation phase includes Rifampicin. |
| ^1^ Timing of ART initiation should be up to clinical judgment based on other signs of immunodeficiency indicating progression of HIV disease (Refer Table 1). For TB patients in WHO clinical Stage IV, ART should be started as soon as TB treatment is tolerated irrespective of CD4 cell count.  ^2^ EFV containing regimens include d4T/3TC/EFV or ZDV/3TC/EFV.  ^3^ NVP (200 mg daily for 2 weeks followed by 200 mg twice daily) may be used in place of EFV in absence of other options. NVP containing regimens include: d4T/3TC/NVP or ZDV/3TC/NVP.  ^5^ Start ART if non-TB Stage IV conditions are present, re-assess the patient for ART at 8 weeks, during the course and at completion of TB treatment. | | |

**References.**

1. Global tuberculosis control: surveillance, planning, financing. WHO report 2005. Geneva, World Health Organization (WHO/HTM/TB/2005.349).

2. Dye C. The global epidemiology of tuberculosis. The Lancet: 2006 -in press.

3. Stop TB Partnership: Global Plan to Stop TB. STOP TB Partnership 2005 retrieved 14/11/05 from:

<http://www.stoptb.org/gpstb/assets/wgstrategicplans/DEWGfull210905.pdf>

4. Dye C, Watts C, Bleed D et al Evolution of Tuberculosis control and prospects for reducing Tuberculosis incidence, prevalence and deaths globally. JAMA 2005; 293: 2767-2775.

5. Elzinga G, Raviglione MC, Dermot Maher. Scale up: meeting targets in global tuberculosis control. Lancet 2004; 363: 814–819.

6. International Union Against Tuberculosis and Lung Disease. 1994. The promise and reality of

fixed-dose combinations with rifampicin. A joint statement of the International Union Against

Tuberculosis and Lung Disease and the Tuberculosis Programme of the World Health

Organisation. Tubercle and Lung Disease, 75 : 180 - 181.

7. Acocella G, Nonis A, Perna G, Patane E, Gialdroni-Grassi G and Grassi C.

Comparative bioavailability of isoniazid, rifampin, and pyrazinamide administered in free

combination and in a fixed triple formulation designed for daily use in antituberculosis

chemotherapy. II. Two-month daily administration. Am Rev Respir Dis 1988, 138 : 886 - 890.

8. Geiter L J, O’Brien R J, Combs D L and Snider Jr. D E. 1987. United States Public Health

Service Tuberculosis Therapy Trial 21 : Preliminary results of an evaluation of a combination

tablet of isoniazid, rifampin and pyrazinamide. Tubercle (Supplement), 68 : 41 - 46.

9. Acocella G.. Human bioavailability studies. Satellite symposium on quality control of

antituberculosis drugs. Bull. IUATLD 1989, 64, 1: 36 - 42

10. Ellard G A, Ellard D A, Allen B A, Girling D J, Nunn A J, Teo S-K, Tan T-H, Ng H-K, and

Chan S-L.. The bioavailability of isoniazid, rifampicin, and pyrazinamide in two

commercially available combined formulations designed for use in the short-course treatment

of tuberculosis. Am Rev Respir Dis 1986. 133 : 1076 - 1080.

11 Blomberg B, Spinaci S, Fourie B, Laing R. The rationale for recommending fixed dose combination tablets for treatment of tuberculosis. Bull WHO 2001;79: 61-8.

12. Hong Kong Chest Service/British Medical Research Council. Acceptability, compliance, and adverse reactions when isoniazid, rifampicin and Pyrazinamide are given as a combined formulation or separately during three-times-weekly antituberculosis chemotherapy. American Review of Respiratory Disease, 1989; 140: 1618-1622.

13. Mitchison DA. How drug resistance emerges as a result of poor compliance during short course chemotherapy for tuberculosis. The International Journal of tuberculosis and Lung Disease, 1998; 2: 10-5
